# Injectable, Adhesive, and Self-Healing Composite Hydrogels Loaded With Oxybutynin Hydrochloride for the Treatment of Overactive Bladder in Rats

**DOI:** 10.3389/fbioe.2022.906835

**Published:** 2022-06-27

**Authors:** Peng Sun, Zheng Wang, Tong Wu, Shishuai Zuo, Xiaoyu Huang, Zilian Cui, Dong Zhang

**Affiliations:** ^1^ Department of Urology, Shandong Provincial Hospital Affiliated to Shandong First Medical University, Jinan, China; ^2^ Department of Chemotherapy, Shandong Second Provincial General Hospital, Shandong Provincial ENT Hospital, Jinan, China; ^3^ Department of Urology, The First Affiliated Hospital of Shandong First Medical University, Jinan, China; ^4^ Department of Urology, Shandong Provincial Hospital, Cheeloo College of Medicine, Shandong University, Jinan, China

**Keywords:** oxybutynin hydrochloride, multifunctional hydrogel, self-healing, wet adhesion, overactive bladder

## Abstract

**Object:** The aim of this study was to prepare injectable, adhesive, and self-healing composite hydrogels loaded with oxybutynin hydrochloride and verify its function in the treatment of overactive bladder.

**Method:** The ultraviolet (UV) absorption of oxybutynin (Oxy) in the solution was detected using a UV spectrophotometer at 233 nm, and the cumulative drug release was calculated using Origin software. L929 mouse fibroblasts were used to test cell adhesion to OCP50 and OCP100 hydrogels. Both FT-IR and NMR overactive bladder demonstrated that Dex was oxidized to PDA with aldehyde groups. Urodynamic examinations were performed 24 h after intraperitoneal injection in the rat model. The relative expression levels of Orai1 and STIM1 were detected by western blot (WB) and QPCR.

**Results:** After loading Oxy, the shear adhesion under the wet conditions of OCP50 and OCP100 was higher than CP50 and CP100 (*p* < 0.05), and both were suitable for intravaginal administration. After 72 h of release, oxybutynin released 82.8% in OCP100 hydrogel and 70% in OCP50. Compared to the model, OCP50, CP100, and OCP100 relieved the overactive bladder and inhibited the expression of Orail and STIM1.

**Conclusions:** Oxybutynin hydrogel could provide relief to overactive bladder by decreasing the expression of Orail and STIM1 in rats.

## 1 Introduction

Overactive bladder (OAB) is a complex symptom, which is characterized by increased urination frequency and urgency, sometimes accompanied by urinary incontinence (Mostafaei et al., 2020). It is a very common disease, especially among the elderly and women, which has a far-reaching impact on the quality of life and is a very heavy burden for health care providers economically (Shiota et al., 2020). The symptoms of OAB can be attributed to the up-regulation of detrusor smooth muscle, which is related to spontaneous and involuntary contraction (Rutman et al., 2021).

The fundamental changes in the electromechanical characteristics of detrusors have been considered as the cause of detrusor overactivity. Meanwhile, the role of intracellular Ca^2+^ is particularly critical since it is the main determinant of detrusor contraction. Storage-operated calcium channels (SOCs) are the main Ca^2+^ entry pathway in several cell types. In SOCs, the consumption of intracellular Ca^2+^ reservoir will stimulate Ca^2+^ to enter from the extracellular environment, and the key molecular determinants of SOCs include STIM1 and Orai1. In this process, STIM1 acts as a Ca^2+^ sensor in the sarco/endoplasmic reticulum, while Orai1 acts as a pore-forming subunit for Ca^2+^ penetration.

In recent years, anticholinergic drugs have been the main treatment for OAB (Song et al., 2021) and oxybutynin hydrochloride is a typical representative. Anticholinergic drugs can block postganglionic muscarinic receptors, reduce bladder contractility, reduce bladder internal pressure, and increase the threshold of urination capacity (Cakir et al., 2019; Escobar et al., 2021). However, in clinical practice, only a few patients insist on using anticholinergic drugs for more than 6 months at present. Oral side effects are the main reasons for stopping the use of OAB drugs (Noh et al., 2019; Burgio et al., 2020). Persistence or compliance with medication is still a major obstacle to the effective management of OAB. In consequence, there is an urgent need for a new method and idea to solve the problems existing in the current clinical treatment of OAB.

Biomaterials are natural or artificial materials used to replace and repair tissues and organs. They have both biocompatibility and functionality and have been widely used in the diagnosis and treatment of a variety of diseases. Among the advanced biomaterial, the hydrogel has been commonly used for drug delivery because of its high-water content and similar natural living tissues (Crane et al., 2018; Qian et al., 2020; Yang et al., 2021).

So in this study, we evaluated the preparation, biocompatibility, and therapeutic effects of oxybutynin hydrochloride–loaded multifunctional hydrogel on OAB model rats. Finally, in this research, we found that the oxybutynin hydrogel could affect the expression of Orail and STIM1 and change the intracellular calcium concentration to improve the progression of the overactive bladder, which provided new methods for the treatment of OAB.

## 2 Materials and Methods

### 2.1 Materials and Reagents

Carboxymethyl chitosan (CMCS) and dextran (Dex) were obtained from Shanghai Yuanye Biotechnology Co., Ltd. (China). Oxy was purchased from AbMole biochemical reagent Co., Ltd., and citric acid and disodium hydrogen phosphate were obtained from Sinopharm Chemical Reagents Co., Ltd. (Shanghai, China). The medium DMEM, fetal bovine serum (FBS,) and 0.25% trypsin/EDTA were purchased from Biological Industries, Israel. 0.5%TritonX-100, DAPI, and rhodamine mark phalloidin was purchased from Beijing Solaibao Technology Co., Ltd. CCK-8 and other reagents were obtained from Sigma-Aldrich Biochemical Technology Co., Ltd.

### 2.2 Instruments and Equipment

PDA50 and PDA100 were characterized using the Fourier-transform infrared spectrometer and 1H NMR (Avance III 500 MHz, Bruker, Germany). CP50, OCP50, CP100, and OCP100 hydrogels were observed by SEM (VEGA3, TESCAN, China). The absorbance of CCK-8 was detected using a microplate reader. The growth of cells was observed using a fluorescence microscope.

### 2.3 Experiments on Animals

#### 2.3.1 Experimental Rats

A total of 30 female SD rats of clean grade, 8 weeks old, were purchased from the Experimental Animal Center of Shandong Province. The feeding conditions of rats are as follows: temperature (22 ± 2)°C, humidity 45%–60%, keeping the circadian rhythm for 12 h, eating and drinking freely, and adaptive feeding for 1 week.

#### 2.3.2 Establishment of the OAB Model

Cyclophosphamide diluted 0.5% solution with normal saline was injected into the rat’s abdominal cavity at a single dose of 200 mg/kg. Urodynamic examination showed that the micturition interval (the time between micturition contractions) was shortened and the micturition threshold (the pressure before micturition contractions) was decreased, which indicated that the model was established successfully.

#### 2.3.3 Experimental Grouping

The rats were randomly divided into five groups: control group (healthy SD rats not treated with any treatment, *n* = 6), model group (OAB model rats, *n* = 6), oxybutynin sustained–release tablet group (OAB model rats, oxybutynin sustained–release tablets were orally taken twice every 24 h, *n* = 6), oxybutynin hydrochloride solution group (OAB model rats were administered vaginally every 48 h, and injected 1 ml solution containing 10 mg Oxy pH-5 phosphate buffer, *n* = 6), and oxybutynin hydrochloride–loaded hydrogel group (OAB model rats, vaginal injection of 1.5 ml OCP100 hydrogel containing 10 mg, *n* = 6).

### 2.4 Preparation and Characteristics of Hydrogels

#### 2.4.1 Synthesis of Oxidized Dextran (PDA)

Oxidized dextran with different degrees of oxidation was synthesized, and the oxidized dextran with the molar ratio of Dex to NaIO_4_ of 1:1 and 2:1 was named PDA50 and PDA100, respectively. Different proportions of dextran and NaIO_4_ were dissolved in deionized water in different molar ratios, stirred in the dark for 24 h, and then dialyzed with distilled water (molecular weight cut-off 8–10 kDa) for 72 h, during which the dialysate must be changed frequently. Finally, the dialyzed original solution was freeze-dried to obtain a white solid, which was stored in a dry condition. The final oxidation degree or aldehyde functionalization of P50 and P100 was 27.68 and 59.49%, respectively.

#### 2.4.2 Synthesis of Oxybutynin Hydrogel (OCP)

A hydrogel containing 10 mg/ml Oxy was prepared. Oxy was dissolved in 5% PDA50 or 5% PDA100 aqueous solution, respectively, to obtain the 20 mg/ml oxybutynin PDA50 solution, and then 10% CMCS aqueous solution was added. The oxybutynin PDA50 or oxybutynin PDA100 solution and CMCS aqueous solution were mixed at a certain ratio (v:v/1:1) to obtain the Oxy hydrogel (respectively indicated as OCP50 and OCP100) containing 10% Oxy. In the control group, Oxy-free hydrogels (indicated as CP50 and CP100) were obtained using the same method.

#### 2.4.3 Hydrogel Adherence

The pigskin was selected as a test for the ability of the hydrogel to adhere to the surface of the human skin. CMCS and OCP100 solution was injected into the surface of the pigskin using a double-mixed suspension needle tube. By changing the shape of the pigskin, we can detect the adhesion of hydrogel on the tissue surface. First of all, add a certain amount of green dye into the PPDA100 solution, use two sterile medical needle tubes to take 1 ml solution of CMCS and PDA50, respectively, and then add a suspension needle to inject it onto the surface of the pigskin. After the gelatin was formed, the shape of the pigskin was changed, and different photographs were taken.

#### 2.4.4 Release of Oxy *In Vitro*

The diffusion of oxybutynin hydrogel was detected *in vitro*. To simulate the *in vivo* environment, the oxybutynin hydrogel was released in a citrate phosphate (pH-5) buffer solution. A measure of 0.8 ml of OCP50 and OCP100 hydrogels were synthesized. They were transferred into dialysis bags (molecular weight cut-off 3,500 Da), and a certain volume of citrate phosphate (pH-5) buffer solution was added into the dialysis bags. They were shaken in a centrifugal tube containing 40 ml of citrate phosphate buffer solution, which was shaken and released at 37°C. Take out 4 ml of the solution at regular intervals, add fresh citrate phosphate buffer, and continue to shake and release at 37°C. The ultraviolet absorption of Oxy in the solution was tested using an ultraviolet spectrophotometer at 233 nm until the release in hydrogel reached equilibrium. The cumulative drug release was counted using Origin software. Three groups of parallel samples were set for each sample.

### 2.5 Biocompatibility Testing

Sterilization treatment of hydrogel material: CMCS, PDA50, and PDA100 solution containing Oxy were irradiated with ultraviolet light for 2 h in an ultra-clean station, and 10% CMCS and PDA50 (or PDA100) solution containing 10 mg/ml Oxy were mixed into the gel with a volume ratio of 1:1 in a ventilated place, then the hydrogel was transferred into a 4 ml DMEM cell culture solution and incubated at 37°C for 24 h.

#### 2.5.1 Cell Culture

L929 mouse fibroblasts were cultured in a cell culture dish containing the DMEM cell culture medium, 10% FBS, and then cultured in a carbon dioxide incubator at 37°C. When the cells grow to more than 90%, subculture was carried out. When the cells were subcultured, the cells were generally digested with 0.05% trypsin/EDTA for 3 min, then the trypsin was sucked out, and the cells were blown by adding a culture medium and transferred to a new sterile culture dish.

#### 2.5.2 Cell Morphology and Viability Assays

To observe whether the hydrogel has an influence on cell growth, the L929 cell nucleus was stained with DAPI, the L929 cell skeleton was stained with rhodamine-labeled phalloidin, and cell density and elongation were observed. L929 cells in a Petri dish were transferred to a 24-well plate with a density of 2×10^4^ cells/well and cultured in a CO_2_ cell incubator. After 24 h, the hydrogel extract was co-cultured with L929 cells in the 24-well plate for 24 h, and the cells were stained with DAPI and rhodamine-labeled phalloidin. The photographs were taken under a fluorescence microscope. The control group was the DMEM culture solution without hydrogel treatment, and the experimental group was DMEM extracts treated with CP50, OCP50, CP100, and OCP100 hydrogels.

Measurement of CCK-8 in L929 cells in a cell culture dish was transferred to a 96-well plate with a cell density of 5×10^4^ cells/well, and cultured in a CO_2_ cell incubator. After 24 h, the hydrogel extract was co-cultured with L929 cells in a 24-well plate for 24 h, then a 10 μl CCK-8 solution was added, and the absorbance of CCK-8 was tested using a microplate reader (OD). Similarly, the setting of the control group and experimental group was the same as cell adhesion.

#### 2.5.3 Urodynamic Examination

The urodynamic examination was carried out 24 h after intraperitoneal injection. SD rats were anesthetized by intraperitoneal injection of 10% chloral hydrate 3 ml/kg. Two epidural catheters were placed through the urethra, one of which was connected with the pressure sensor of the BL-420 biological function experimental system, and the other was connected with a micro-perfusion pump. Normal saline was injected at a rate of 0.2 ml/min. The micturition interval, basal bladder pressure, and bladder pressure were measured and recorded.

#### 2.5.4 Western Blot

Tissue pieces were washed with cold PBS to remove blood stains, cut into small pieces, and put into homogenization tubes. Add 1–22 mm magnetic beads, add 10 times the tissue volume of this reagent, and put it in a homogenizer, and select the program to homogenize thoroughly. Take out the homogenized sample tube, ice bath for 30 min, and shake once every 5 min to ensure that the tissue is completely cracked. Centrifuge at 12000 rpm for 10 min, and collect the supernatant. The BCA protein concentration determination kit was used to determine the protein concentration of the sample. The protein samples were separated by 12% SDS-PAGE and transferred to the PVDF membrane. The membrane was blocked with TBS containing 5% BSA for 2 h. The second antibody coupled with horseradish peroxidase showed immune response bands. ImageJ software was used to quantify the intensity of immunoblot bands.

#### 2.5.5 QPCR

Total RNA was extracted using the TRIzol reagent (Invitrogen, CA, United States). Reverse transcription was performed in a 20 μl reaction mixture, and cDNA was synthesized from 2 μg total RNA. The gene expression of the prepared cDNA was further analyzed by real-time RT-PCR by gene-specific primers. Real-time PCR was carried out with Bio-Rad SSO Fast Evageleen Supermix. The cycle conditions are as follows: 94°C for 5 min, and the following procedures were used for 45 cycles: 94°C 30 s, 54.3°C 30s, and 72°C 45s. RNA abundance was expressed by ^△△^Ct, and the fluorescence signal expressed by the target gene was normalized to that of the internal control (GAPDH). Primer sequences: Orai1 5′- ACG​TCC​ACA​ACC​TCA​ACT​CC -3′; 5′-GGT​ATT​CTG​CCT​GGC​TGT​CA -3'; STIM1 5′- GGC​CAG​AGT​CTC​AGC​CAT​AG -3′ 5′- TAG TCGCACCTCCTGG ATAC -3′; GAPDH 5′- TCACCATC TTCCAGGAGCGA -3′ 5′-TGCTGGTGAAGCC GTAACAC-3'.

### 2.6 Statistical Analysis

All values are expressed as mean ± SD. Paired or unpaired Student’s t-test was used to determine significant differences for comparison between two groups, or use one-way ANOVA, Newman–Keuls test for comparison among multiple groups, or use two-way ANOVA for the Bonferroni *post hoc* test for comparison among multiple groups. All the analyses were carried out using GraphPad Prism software (Graphpad Software, Inc., San Diego, CA, United States). When *p* < 0.05, the difference was considered statistically significant.

## 3 Results

### 3.1 Synthesis of PDA With Different Oxidation Degrees

**FIGURE 1:**
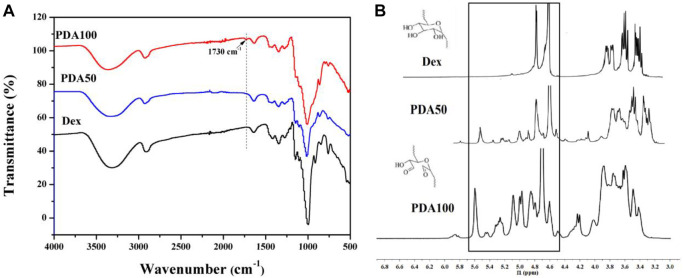
**(A)** FT-IR spectrum Dex, PDA50, and PDA100; **(B)** 1H NMR spectrum of Dex, PDA50, and PDA100.

By FT-IR and NMR characterization of Dex and PDA, it can be seen that we successfully oxidized Dex to obtain dextran with the aldehyde group. As shown in Figure 1A, the infrared absorption peaks of Dex, PDA50, and PDA100 are similar, and PDA100 had an obvious vibration absorption peak at 1730 cm^−1^. However, the absorption peak of PDA50 at 1730 cm^−1^ was weak, which was due to the proportion of oxidized dextran synthesized. Because the proportion was obvious, PDA had an absorption peak, while the absorption peak of PDA50 was not obvious at 1730 cm^−1^. Therefore, we successfully synthesized oxidized dextran. As shown in Figure 1B, PDA50 and PDA100 produced a large amount of hemiacetal between 4.4 and 5.8 ppm. FT-IR and NMR both proved that Dex was oxidized to PDA with the aldehyde group. (Figure 1).

### 3.2 Synthesis and Characterization of OCP Hydrogels

**FIGURE 2:**
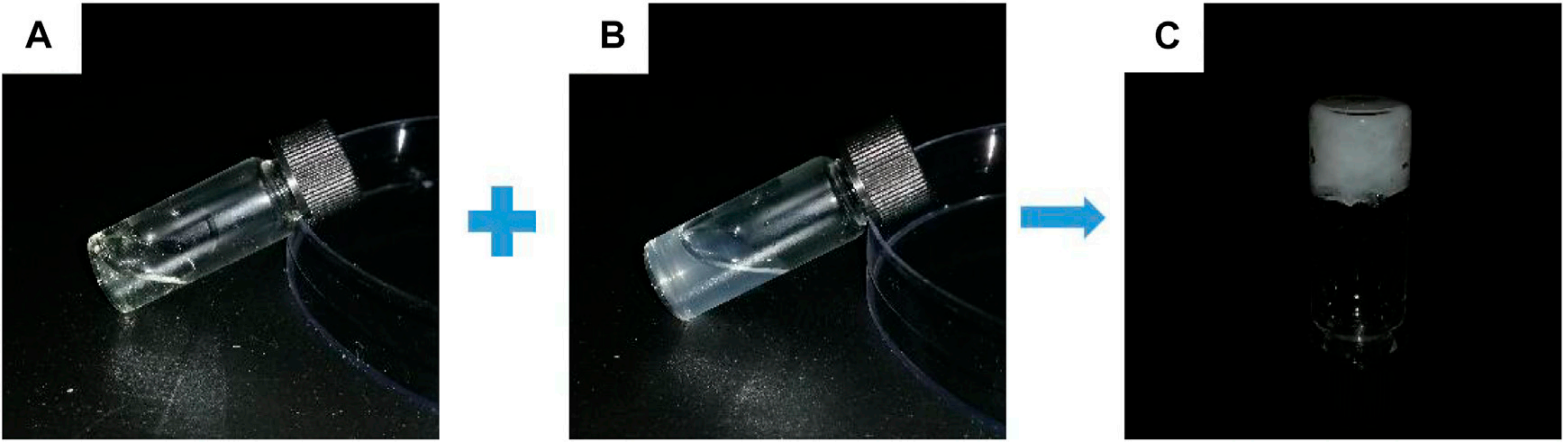
Gel-forming picture of the hydrogel. **(A)** CMCS aqueous solution **(B)** Oxy PDA100 aqueous solution and **(C)** OCP100 hydrogel.

At room temperature, it was easy to prepare hydrogels by mixing the CMCS aqueous solution with PDA50 or PDA100 solution loaded with Oxy within 8s through the dynamic Schiff base reaction. (Figure 2).

### 3.3 Characterization of OCP Hydrogels

**FIGURE 3:**
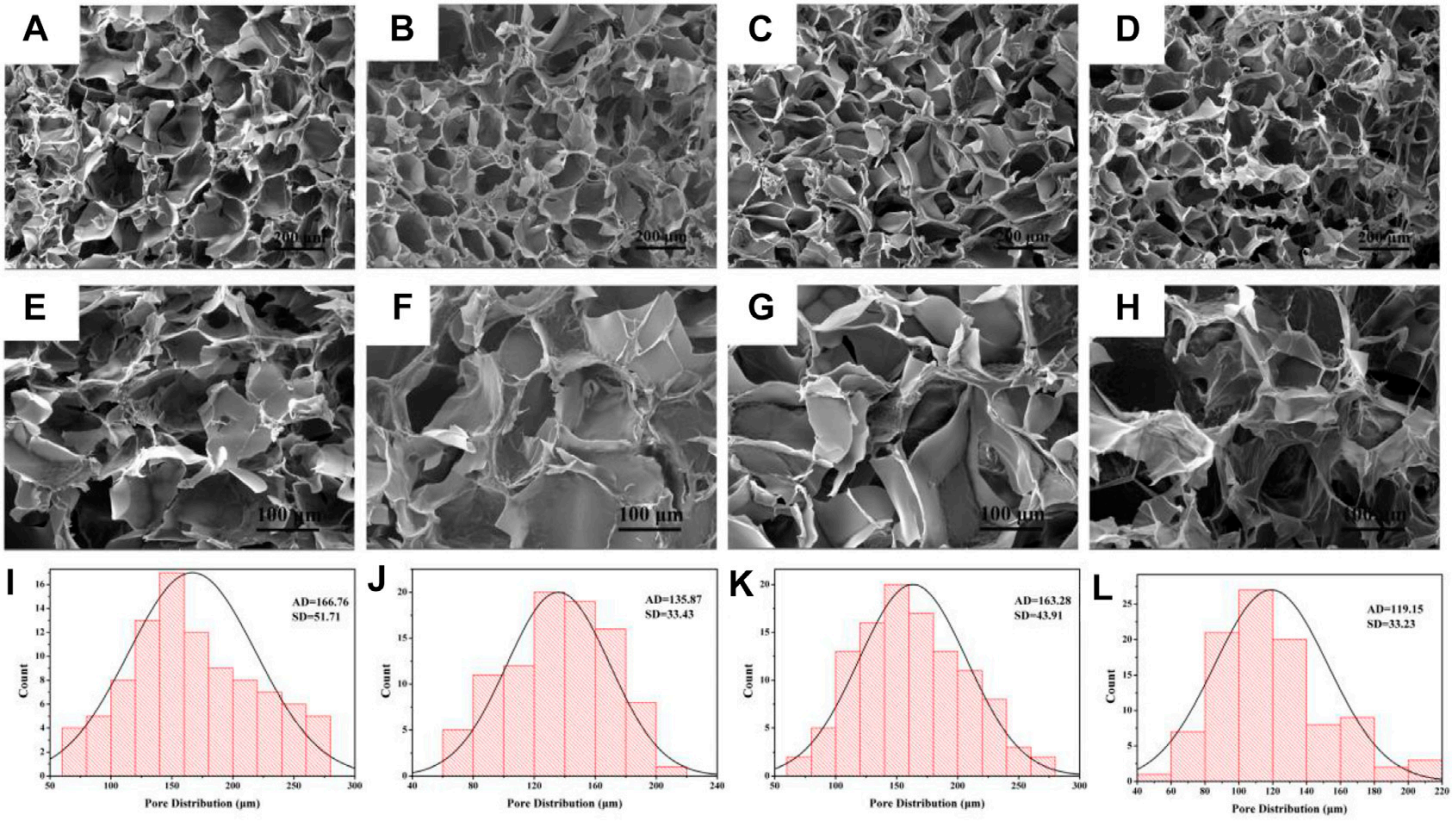
Morphology and diameter distribution of hydrogel. **(A–D)** SEM images of CP50, OCP50, CP100, and OCP100 hydrogels (200×); **(E–H)** SEM images of CP50, OCP50, CP100, and OCP100 hydrogels (500×); **(I–L)** Pore statistics of cross-section of CP50, OCP50, CP100, and OCP100 hydrogels.

Hydrogels were formed in aqueous solution by 1:1 reaction of CMCS solution and Oxy PDA. After the freeze-dried hydrogel was cut transversely, it was observed by SEM. As shown in Figures 3A–D, the pore size of each hydrogel possessed interconnected porous structures with relatively homogeneous distribution. By magnifying 500 times (Figure 3E–G), it can be seen that the surface of the pores was smooth, indicating that the Oxy drug was dissolved in the PDA solution. According to the statistics of the pore sizes of hydrogels in SEM images, the pore sizes of hydrogels formed by PDA50 (CP50 and OCP50) were 166.76 ± 51.71 and 135.87 ± 33.43 μm, respectively, and those formed by PDA100 (CP100 and OCP100) were 163.28 ± 43.91 and 119.15 ± 33.23 μm, respectively (Figures 3 I–L). From the aforementioned data, it can be seen that the aperture formed by PDA50 and PDA100 after loading Oxy becomes slightly decreased. This was beneficial to the release of Oxy *in vivo* (Figure 3).

### 3.4 Adhesion Test of OCP Hydrogel

**FIGURE 4:**
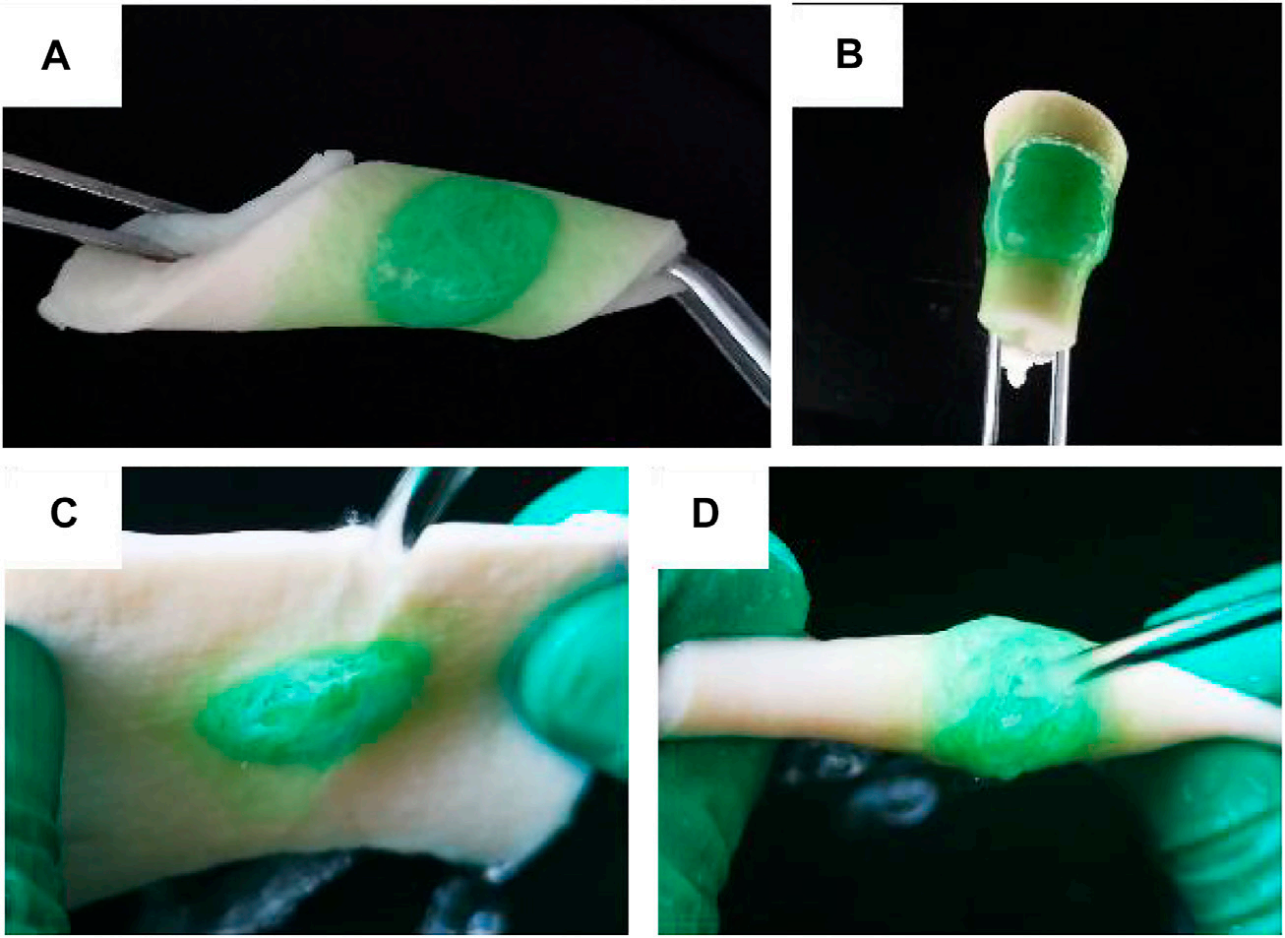
Photo of OCP100 hydrogel adhered to the pigskin. **(A)** Side bending, twisting, and curling; **(B)** folding and twisting; **(C)** lateral water flow impact; **(D)** double water flow impact.

To verify the wet adhesion of OCP100 hydrogel, the OCP100 hydrogel was injected into the pigskin surface through a double mixing needle tube, and the hydrogel could be firmly attached to the pigskin surface by different twisting ways, such as side bending (Figure 4A) and double bending (Figure 4B). Cervical mucus could flow out from the vagina. To detect the influence of mucus in the vagina on hydrogel adhesion, the hydrogel adhered to the pigskin was placed under the faucet, and the hydrogel OCP100 adhered to the pigskin surface was impacted by a rapid water flow (Figures 4C,D). The OPC100 hydrogel did not break or fall off, and can be firmly adhered to the pigskin surface. Therefore, the OCP100 hydrogel can firmly adhere to the surface of the vaginal wall *in vivo*. In the adhesion test, it was found that the shear adhesion of oxybutynin-loaded hydrogels (OCP50 and OCP100) was higher than that of oxybutynin-loaded hydrogels (CP50 and CP100) (Figure 4E). In the experiment, the hydrogel was injected on the surface of the plastic board with pigskin attached, and two pigskins were bonded together by the hydrogel. Under the action of universal dynamometer pulling force, the two pigskins were separated, and the hydrogel loaded in the middle was broken. The hydrogel was bonded for 20 min to test the pulling force. Through the shear adhesion test, the shear adhesion of OCP50 and OCP100 was higher than that of CP50 and CP100 after loading Oxy (*p* < 0.05). (Figure 4).

### 3.5 *In Vitro* Release of Drug-Loaded Hydrogel

**FIGURE 5:**
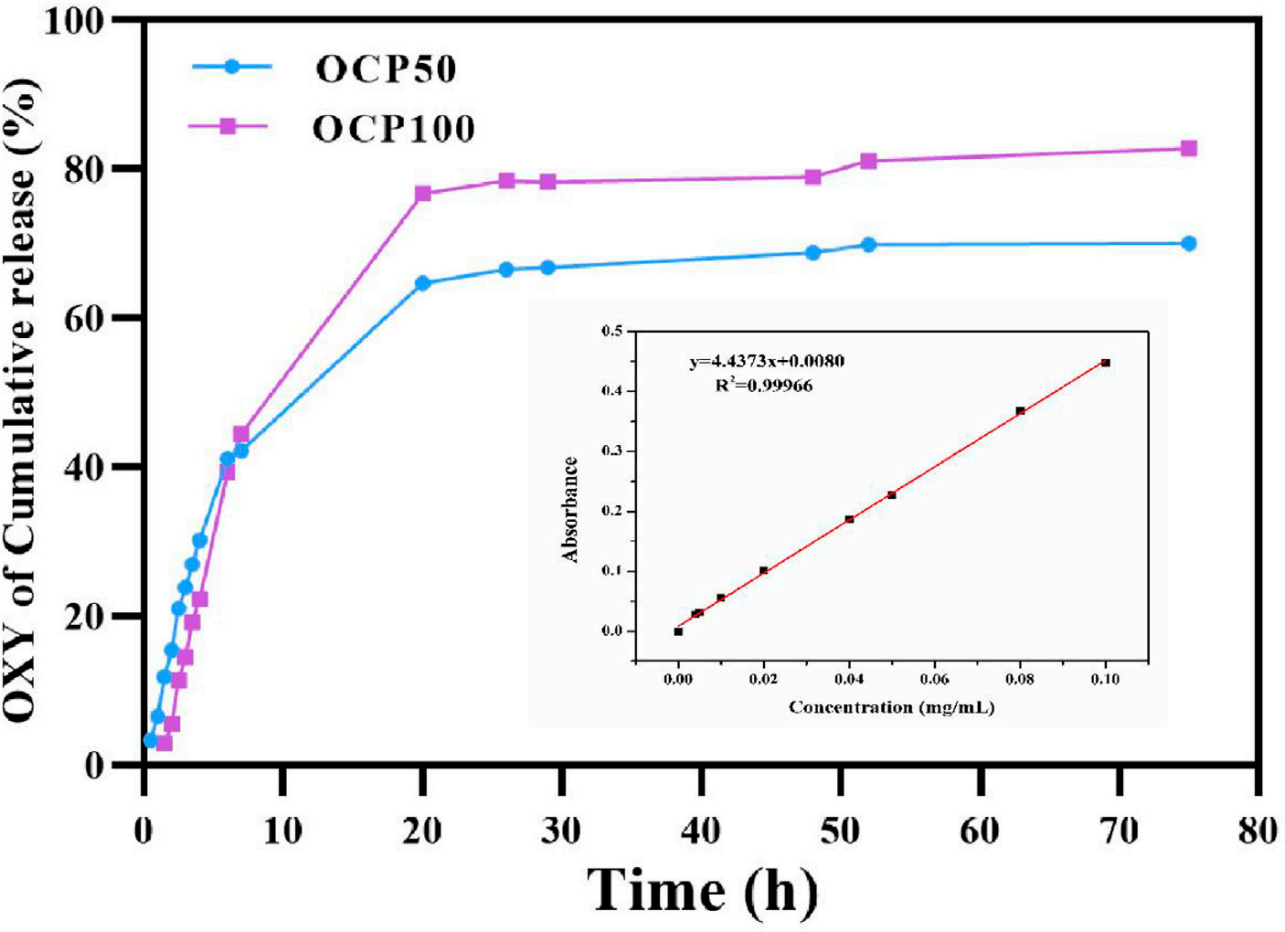
*In vitro* drug release of OCP hydrogel in the acidic buffer solution.

To simulate the release of hydrogel *in vivo*, the hydrogel was released in a citrate phosphate buffer solution (pH-5) at 37°C *in vitro*. The release of oxybutynin hydrogel in a buffer solution showed that the oxybutynin hydrogel had a rapid release within 0–10 h, which may be due to the higher initial release rate of through osmosis. After 20 h, the slope of release decreased and the release rate decreased, which may be difficult to diffuse into the buffer solution of the oxybutynin inside the hydrogel. After 72 h of release, oxybutynin released 82.8% in the OCP100 hydrogel and 70% in OCP50, so PDA did not affect the diffusivity of Oxy (Figure 5). PDA with a deeper oxidation degree had the better release of Oxy likely due to the loose structure of OCP100 (Figure 3H).

### 3.6 Biocompatibility Test

**FIGURE 6:**
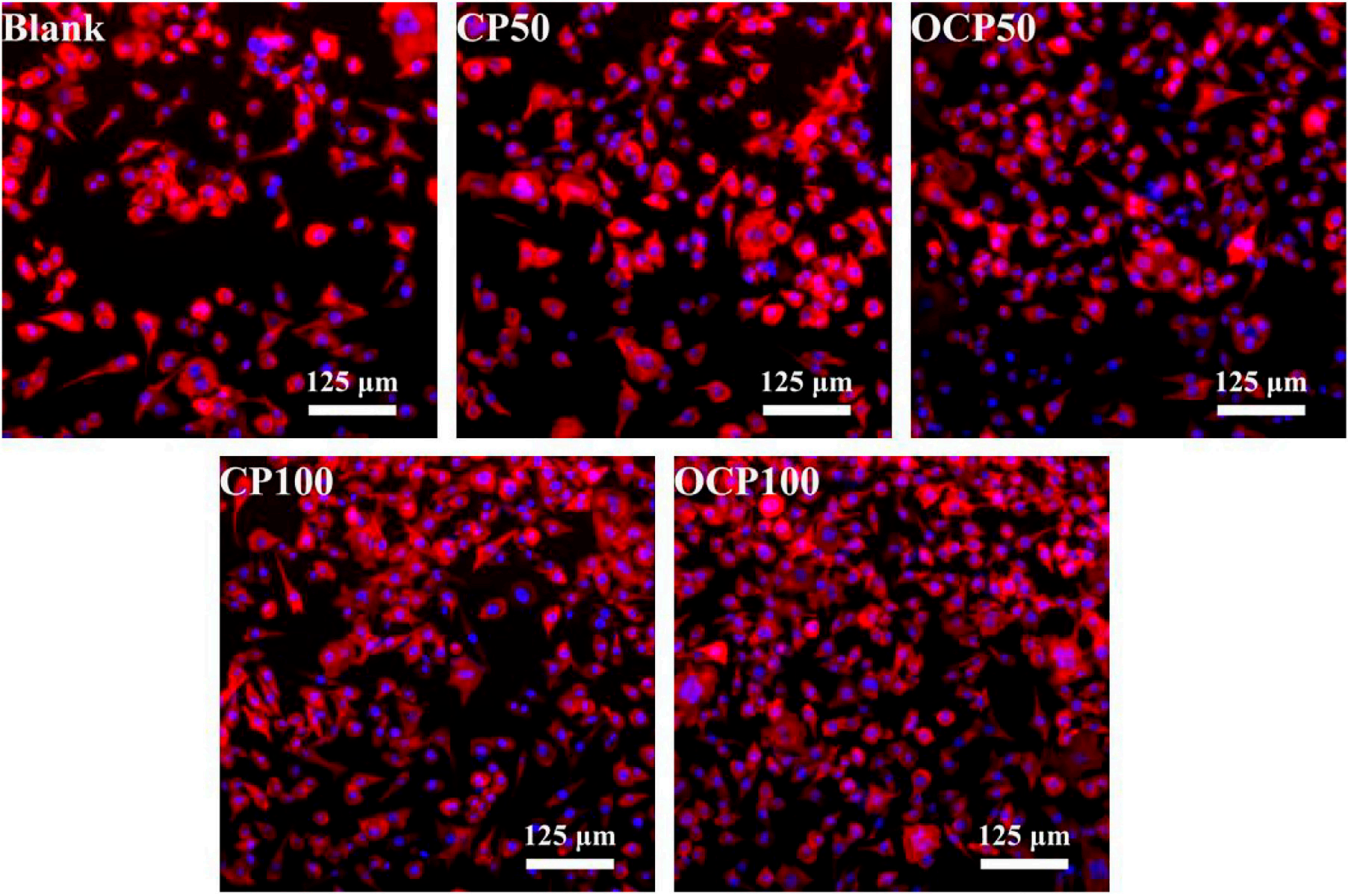
Adhesion morphology of cells after co-culture of hydrogel and cells.

To verify the toxicity of materials to cells, L929 mouse fibroblasts were used to test the cell response of OCP50 and OCP100 hydrogels. After incubating with cell hydrogel extracts for 24 h, in comparison with the control group, the cell nucleus and cytoskeleton were stained, and the cell morphology was better after incubating cells with CP50, CP100, OCP50, and OCP100 hydrogel extracts Figure 6.

The quantitative results of cells showed that the cell density of OCP50, CP100, and OCP100 was higher than that of the blank group (*p* < 0.05). (Table 1).

**TABLE 1:** Cell density and elongation.

Group	Cell density (number of cells mm^2^)	Cell elongation	CCK-8 assay
Blank	22.87 ± 1.95	2.03 ± 0.89	1.41 ± 0.06
CP50	25.34 ± 2.48	2.05 ± 1.04	1.48 ± 0.11^c^
OCP50	32.65 ± 5.56^a^	1.76 ± 1.12	1.53 ± 0.06^a^
CP100	38.21 ± 1.05^b^	2.02 ± 1.87	1.56 ± 0.03^b^
OCP100	41.89 ± 2.18^b^	1.83 ± 0.95	1.59 ± 0.08^b^

a *p* < 0.05 compared with blank.

b *p* < 0.01 compared with blank.

The CCK-8 assay was used to test the toxicity of extracts of each group to cells. In comparison with the blank group, the OD values of OCP50, CP100, and OCP100OD in the experimental group were higher, and the cell survival was better. Therefore, the hydrogel had excellent cytocompatibility for application as a biomaterial-based drug delivery system.

### 3.7 Urodynamic Examination

In comparison with the control group, the time limit of micturition interval in the model group was shorter (*p* < 0.05), the basal bladder pressure was reduced (*p* < 0.05), the micturition threshold was raised (*p* < 0.05), and the average detrusor pressure was increased (*p* < 0.05). (Table 2).

**TABLE 2:** Cell proliferation (S) was detected by MTT assay.

Group	Only a Few	Urination interval/s	Basal bladder pressure/cmH_2_O	Urination threshold/cmH_2_O	Mean detrusor pressure/cmH_2_O
Control group	3	421.58 ± 102.48	15.46 ± 0.99	7.48 ± 1.01	43.62 ± 2.43
Model group	12	177.86 ± 74.71	10.25 ± 1.43	11.94 ± 1.98	54.45 ± 12.52
*T-value*		0.992	3.171	1.163	0.299
*p-value*		<0.001	<0.001	0.003	0.170

### 3.8 WB Detection of the Relative Expression of Orai1 and STIM1 Protein

**FIGURE 7:**
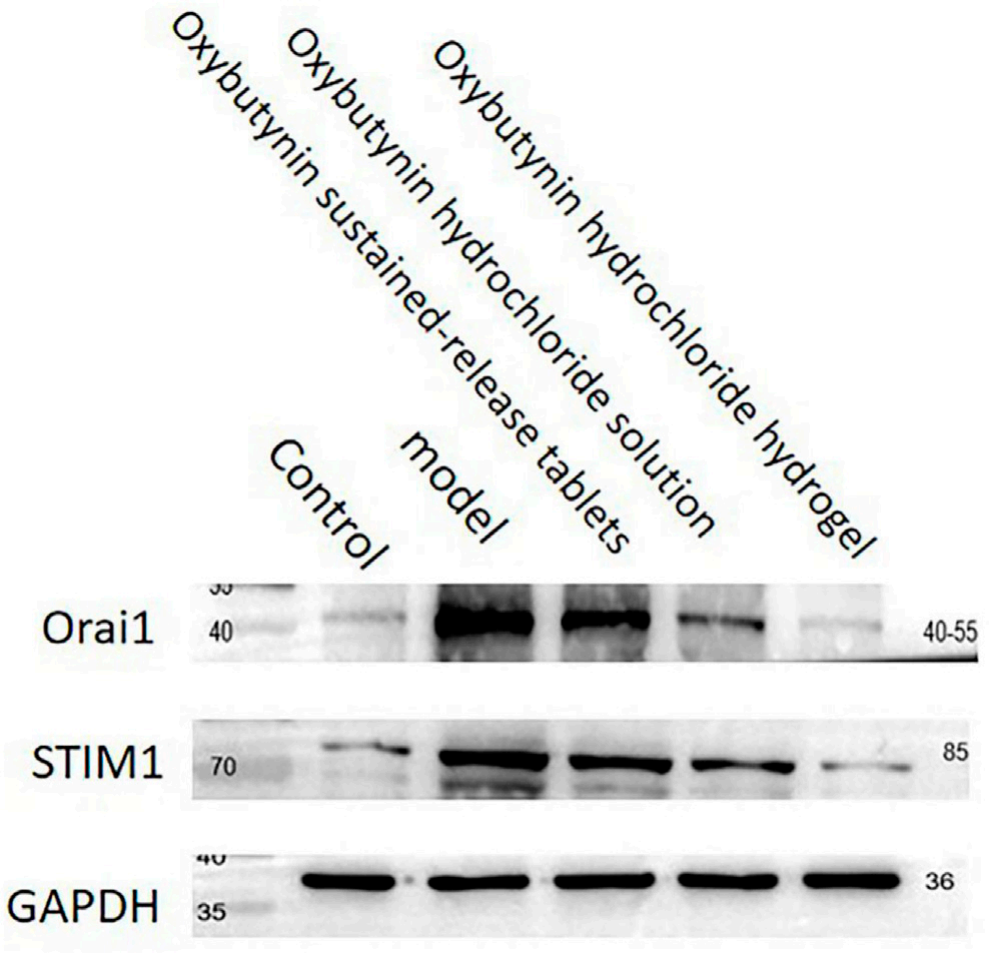
Relative expression of oral mRNA and STIM1 mRNA.

The relative expression of Orai1 and STIM1 protein was tested by WB. In comparison with the control group, the protein expression of Orai1 and STIM1 in the model group, oxybutynin sustained–release tablet group, and oxybutynin hydrochloride solution group increased (*p* < 0.05), but there was no significant difference in the oxybutynin hydrochloride hydrogel group (*p* > 0.05). In comparison with the model group, the protein expression of Orai1 and STIM1 in the oxybutynin sustained–release tablet group, oxybutynin hydrochloride solution group, and oxybutynin hydrochloride hydrogel group decreased (*p* < 0.05), and the oxybutynin hydrochloride hydrogel group decreased the most. (Figure 7).

### 3.9 QPCR Test to Test the Relative Expression of Oral mRNA and STIM1 mRNA

**FIGURE 8:**
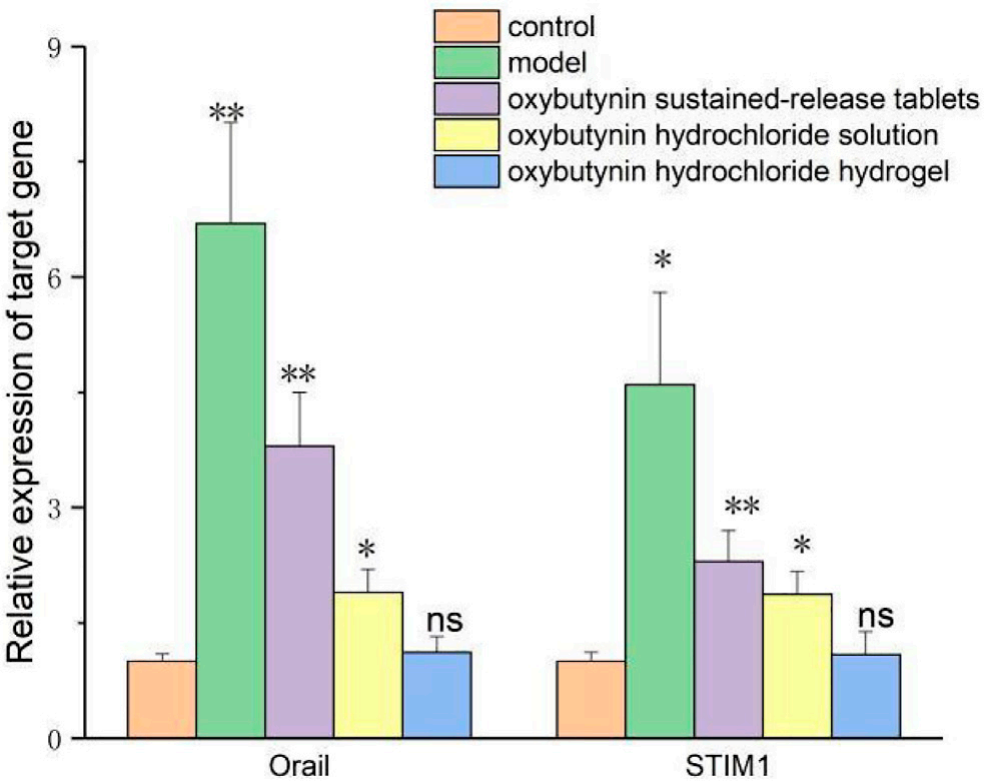
Relative expression of oral mRNA and STIM1 mRNA. *meansP<0.05, **means *p* < 0.01, ns means not significant.

The relative expression of Orail and STIM1 mRNA was detected by the QPCR test. In comparison with the control group, the relative expression of Orail and STIM1 mRNA in the model group, oxybutynin sustained–release tablet group, and oxybutynin hydrochloride solution group increased (*p* < 0.05), but there was no significant difference in oxybutynin hydrochloride hydrogel group (*p* > 0.05). In comparison with the model group, the relative expressions of Orail mRNA and STIM1 mRNA in the oxybutynin sustained–release tablet group, oxybutynin hydrochloride solution group, and oxybutynin hydrochloride hydrogel group decreased (*p* < 0.05), and the oxybutynin hydrochloride hydrogel group decreased the most. (Figure 8).

## 4 Discussion

OAB is defined as urgency with or without urgent urinary incontinence, and the prevalence of OAB increases with age (Welk and McArthur, 2020). OAB with urgent urinary incontinence has an important impact on the economic burden of patients and the medical care system, and on public health and clinical management (Wang et al., 2019; Staskin et al., 2020; Dmochowski et al., 2021). Many oxybutynin preparations have been developed, including various oral preparations and solutions (Ramsay et al., 2020; Vozmediano-Chicharro et al., 2020; Suzuki et al., 2021). The first developed immediate-release oral preparation has high side effects. Compared with the immediate release, the introduction of oxybutynin sustained–release oral preparation reduced the incidence of anticholinergic events, thus improving the compliance of oral oxybutynin. However, sustained-release preparations are still associated with a high incidence of xerostomia. Pharmacokinetic evidence shows that the anticholinergic adverse reaction of oxybutynin is mainly attributed to the high plasma concentration of N- diethyloxybutynin (N-DEO). N-DEO is produced in large quantities in the liver and intestine through the first-pass metabolism of oral oxybutynin involving cytochrome P-450. The plasma concentration of N-DEO produced by immediate-release oxybutynin may be as high as 10 times to that of oxybutynin itself. In comparison with immediate-release oxybutynin, the improvement of anticholinergic adverse events of sustained-release oxybutynin is attributed to the decrease in the ratio of total N-DEO to oxybutynin plasma exposure. For sustained-release preparations, N-DEO and oxybutynin seem to have a similar affinity for the muscarinic M3 receptor, which represents the main receptor subtype in salivary glands and is thought to mediate bladder smooth muscle contraction during urination.

The vaginal route is an alternative method for women who cannot tolerate the side effects of oral antimuscarinic drugs or the application site reactions related to topical/transdermal products. Generally speaking, the biodegradation rate of polysaccharides is relatively fast. There are many reports about chitosan cross-linking to form a hydrogel, which usually involves small molecular cross-linking agents, but they have cytotoxic potential. In addition, the slow gelation of some formulations makes them impractical for applications requiring rapid *in situ* gelation. Dextran is a naturally occurring bacterial polysaccharide, which is mainly composed of *α*-1,6- linked d-glucopyranose residues and a small amount of *α*-1,2, *α*-1,3, or *α*-1,4-linked side chains. Because of its biodegradability and non-toxicity, dextran has been used as a macromolecular carrier for the delivery of drugs or proteins and the separation and purification of biomaterials. Oxidizing dextran with periodate is a classical method of dextran functionalization, which produces entities with multiple functional aldehyde groups, and it can be used as macromolecular cross-linking agents for those polymers with free amino groups to form hydrogels. After loading Oxy, the shear adhesion of OCP50 and OCP100 was higher than that of CPP 100, which indicated that the adhesion of OCP50 and OCP100 was stronger after loading drugs, and both are suitable for vaginal administration. Within 0–10 h, oxybutynin was released rapidly in the hydrogel, which may be due to the higher initial release rate of oxybutynin through osmosis. After 20 h, the slope of release decreases and the release rate decreased, which may be difficult to diffuse into the buffer solution of the oxybutynin inside the hydrogel. After 72 h of release, oxybutynin released 82.8% in OCP100 hydrogel and 70% in OCP50, so PDA did not affect the diffusivity of oxy. PDA with a deeper oxidation degree had the better release of Oxy. After incubation with the cell hydrogel extract for 24 h, the biocompatibility of hydrogel to L929 cells was good. Researchers are increasingly finding a way to make damaged hydrogels heal themselves by mechanically creating a “side-suspended polymer chain” that dangles from the main structure of the hydrogel, giving the damaged parts a chance to mount and reattach (Bai et al., 2020; Höglinger et al., 2021). The scientists also used computer simulations to show that the length of the strand is crucial to the healing process of damaged hydrogels, with the right length enhancing their ability to repair themselves. The self-healing hydrogel showed good biocompatibility with the bladder before and after self-healing, indicating its potential biomedical value in the treatment of bladder injury ^[22]^.

The increase of intracellular Ca^2+^ is characterized by Ca^2+^ entering through SOCs, which is very important for particle release. The regulation of SOC activity involves many mechanisms. STIM1 and Orai1 play a key role in the signal conduction and infiltration mechanism of Ca^2+^ inflow through SOCs ^[1,923,204]^. The overexpression of STIM1 and Orai1 leads to a significant increase of Ca^2+^ entry during storage operation in cells. When calcium storage is exhausted, the N-terminal of STIM1 is glycosylated and transferred from the endoplasmic reticulum to the cell membrane, which is a necessary process to activate SOCs ^[25–27]^. In this study, it was found that the levels of Orai1 and STIM1 increased in OAB model rats, which was considered as an important reason for SOCs.

To sum up, dextran oxidized by periodate can be used as a macromolecular cross-linking agent of oxybutynin multifunctional hydrogel, which showed sustained drug release curve *in vitro* research, had stronger adhesion and better biocompatibility to cells, and was suitable for vaginal administration. Oxybutynin hydrochloride hydrogel mediated Ca^2+^ entry by increasing the relative expressions of Orail mRNA, STIM1 mRNA, and protein in OAB rats, which improved the therapeutic effect compared with oxybutynin sustained–release tablets and oxybutynin hydrochloride solution, and was a promising new therapy for OAB.

## Data Availability

The original contributions presented in the study are included in the article/Supplementary Material; further inquiries can be directed to the corresponding author.
